# Medium chain length polyhydroxyalkanoates as potential matrix materials for peripheral nerve regeneration

**DOI:** 10.1093/rb/rbad063

**Published:** 2023-07-21

**Authors:** Rinat Nigmatullin, Caroline S Taylor, Pooja Basnett, Barbara Lukasiewicz, Alexandra Paxinou, Lorena R Lizarraga-Valderrama, John W Haycock, Ipsita Roy

**Affiliations:** Higher Steaks Ltd., 25 Cambridge Science Park Rd, Milton, Cambridge CB4 0FW, UK; School of Life Sciences, College of Liberal Arts and Sciences, University of Westminster, London W1B 2HW, UK; Department of Materials Science & and Engineering, The University of Sheffield, Sheffield S3 7HQ, UK; School of Life Sciences, College of Liberal Arts and Sciences, University of Westminster, London W1B 2HW, UK; School of Life Sciences, College of Liberal Arts and Sciences, University of Westminster, London W1B 2HW, UK; School of Life Sciences, College of Liberal Arts and Sciences, University of Westminster, London W1B 2HW, UK; Foundation of Research and Technology Hellas, Institute of Chemical Engineering and High Temperature Chemical Processes (FORTH/ICE-HT), P.O. Box 1414, GR 26504, Rion, Patras, Greece; School of Life Sciences, Queen's Medical Centre, University of Nottingham, Nottingham NG7 2UH, UK; Department of Materials Science & and Engineering, The University of Sheffield, Sheffield S3 7HQ, UK; Department of Materials Science & and Engineering, The University of Sheffield, Sheffield S3 7HQ, UK

**Keywords:** peripheral nerve injury, nerve regeneration, biomaterials, mcl-polyhydroxyalkanoates, Schwann cells, NG108-15

## Abstract

Polyhydroxyalkanoates are natural, biodegradable, thermoplastic and sustainable polymers with a huge potential in fabrication of bioresorbable implantable devices for tissue engineering. We describe a comparative evaluation of three medium chain length polyhydroxyalkanoates (mcl-PHAs), namely poly(3-hydroxyoctanoate), poly(3-hydroxyoctanoate-co-3-hydoxydecanoate) and poly(3-hydroxyoctanoate-co-3-hydroxydecanoate-co-3-hydroxydodecanoate), one short chain length polyhydroxyalkanoate, poly(3-hydroxybutyrate), P(3HB) and synthetic aliphatic polyesters (polycaprolactone and polylactide) with a specific focus on nerve regeneration, due to mechanical properties of mcl-PHAs closely matching nerve tissues. *In vitro* biological studies with NG108-15 neuronal cell and primary Schwann cells did not show a cytotoxic effect of the materials on both cell types. All mcl-PHAs supported cell adhesion and viability. Among the three mcl-PHAs, P(3HO-*co*-3HD) exhibited superior properties with regards to numbers of cells adhered and viable cells for both cell types, number of neurite extensions from NG108-15 cells, average length of neurite extensions and Schwann cells. Although, similar characteristics were observed for flat P(3HB) surfaces, high rigidity of this biomaterial, and FDA-approved polymers such as PLLA, limits their applications in peripheral nerve regeneration. Therefore, we have designed, synthesized and evaluated these materials for nerve tissue engineering and regenerative medicine, the interaction of mcl-PHAs with neuronal and Schwann cells, identifying mcl-PHAs as excellent materials to enhance nerve regeneration and potentially their clinical application in peripheral nerve repair.

## Graphical abstract

**Figure:**
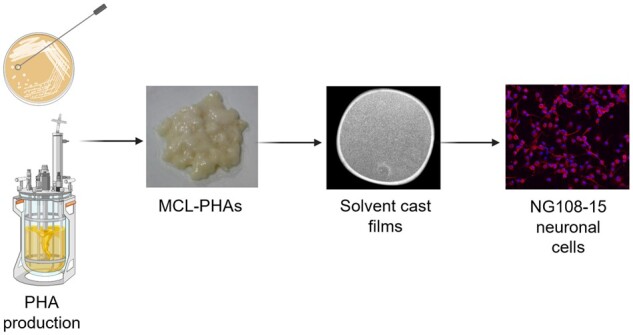
Created in Biorender.com

## Introduction

Peripheral nerve injuries (PNI) have become a global problem affecting over 20 million people every year only in the USA [1]. Peripheral nerve damages are mainly caused by severe physical injuries, iatrogenic injuries, or tumours [2]. PNI have a great impact on the daily life of patients by creating serious difficulties with everyday activities and mobility or leading to lifelong disabilities [3]. Even with some level of potential for the self-regeneration of the peripheral nervous system (PNS), nerve injuries result in the substantial loss of neuronal communication, as well as significantly limited restoration of the motor and sensor function [4]. In general, for nerve gaps below 2 cm, the level of neuronal regeneration could be moderate, whereas for nerve gaps >4 cm, regeneration is almost impossible. Therefore, the complexity of nerve regeneration continues to be a great challenge for the nerve reconstruction and repair [5]. The critical parameters, which affect the peripheral nerve regeneration process, include the size of the nerve gap, time between injury and applied treatment as well as the patients’ medical condition [6]. The traditional treatment of PNI include end-to-end tensionless suturing, allotransplantation and auto-transplantation [7]. The current gold standard techniques of using nerve allografts and autografts are associated with serious drawbacks, such as donor site morbidity, limitation of donor tissue and the necessity of multiple surgeries, as well as immunological rejection when using allografts [8]. Presently, AxoGen Avance^®^ is the only human allograft with Food and Drug Administration (FDA) approval for the peripheral nerve reconstruction [9]. Therefore, there is a high demand for alternative solutions with significant effort on the development of artificial nerve guidance conduits (NGCs), hollow cylindrical structures fabricated mainly from various types of polymeric materials. The main role of the NGCs is to provide a specific microenvironment for effective nerve regeneration by guiding new forming axons across the gap, isolating and protecting newly re-developed nerves from the negative impact of the surrounding environment [10]. An ideal material to produce a NGC needs to demonstrate specific properties, such as flexibility, permeability, swelling and an appropriate degradation rate [11]*.* Both synthetic and natural polymers, such as silicone, poly(D, L-lactide-*co*-ε-caprolactone) (PLCL), polyvinyl alcohol, polyglycolic acid, collagen and chitosan have been explored for their suitability for the development of NGCs [9]. Silicone tubes were used as the first artificial nerve conduits in the 1980s by Lundborg *et al*. [12] to repair rat sciatic nerves with gaps <1 cm. However, lack of degradability of the material caused an inflammatory response and required additional surgery for the tube removal.

Additionally, non-degradable materials can lead to compression neuropathy or accelerated fibrotic reaction [6]. Therefore, the biodegradable materials have been considered as a feasible option to overcome the limitations of the non-degradable NGCs. Although, many natural polymers, such as collagen, chitosan, silk and alginate, have been used in neuronal tissue engineering, the weak mechanical properties, alongside thermal sensitivity and challenges during processing enforced the addition of the synthetic polymers to overcome these material limitations. On the other hand, the application of only synthetic polymers often requires an addition of natural polymers to enhance material biocompatibility [13]. The most extensively used materials, with FDA approval for artificial nerve grafts, are collagen commercialized under tradenames NeuraGen^®^, Neuroflex^TM^, NeuroMatrix^TM^, NeuraWrap^®^, NeuroMend^TM^ (made from collagen type I) and PLCL (Neurolac^®^ and Neurotube^®^) [6, 9]. The main limitations of the biomaterials currently used for NGCs are related to the inappropriate degradation rate. Collagen conduits disintegrate before complete nerve regeneration occurs, whereas slow, incomplete adsorption of PLCL conduits results in unwanted immune responses with reports indicating early collapse of Neurolac^®^ PLCL conduits [9].

Moreover, there is an increased risk of the occurrence of an inflammatory response and toxic effect of degradation products. Most of the biomaterials have been more successful in small nerve defects [6]. This necessitates a demand for novel resorbable biomaterials supporting peripheral nerve regeneration. Simplicity and robustness of hydrolytic degradation under physiological conditions prompted the search of suitable polymers for NGCs among polyesters. Polyhydroxyalkanoates (PHAs) are thermoplastic, aliphatic polyesters of natural origin and have widened the selection of resorbable polymers suitable for biomedical applications. PHAs are the broad family of the natural polymers, mainly produced by bacterial strains under specific conditions during a fermentation process [14, 15]. Thermoplastic properties, are desirable for NGCs production, as then can be easily processed to form a patent structure. These linear bio-polyesters demonstrate beneficial biological features, such as proven high biocompatibility for a wide range of cell lines, biodegradation via surface erosion into weakly acidic and non-toxic products, as well as versatile mechanical and thermal properties, which makes them suitable candidates for the reconstruction and regeneration of injured nerves [16]. The chemical composition of PHAs can be tuned by using different types of carbon sources in the fermentation process. In general, PHA properties are mainly driven by the number of carbon atoms within the monomer unit. PHAs also offer many advantages over conventional biopolymers, due to their green origins, produced by bacteria, and their sustainability, having been shown to be produced using waste food products as a carbon source [17]. Therefore, PHAs can be divided into two main groups: short chain length PHAs (scl-PHAs) with the number of carbon atoms from 3 to 5 within the monomer unit and medium chain length PHAs (mcl-PHAs) with 6–14 carbon atoms within the monomer unit [16, 17]. The main material properties of polymers from the scl-PHA group are high stiffness and strength, brittleness and high crystallinity. Whereas mcl-PHAs are elastomeric and flexible materials, low melting point and low crystallinity in comparison to scl-PHAs [14]. PHAs by several tens of degrees, the amorphous part of all PHAs is in a rubbery state at room and body temperatures. The broad range of material properties along with high biocompatibility and biodegradability makes the PHAs highly promising candidates for various biomedical applications. PHAs have been successfully used in drug delivery, hard and soft tissue engineering, biomedical device development and nerve regeneration [18].

Amongst various types of PHAs, poly(3-hydroxyburyrate), known as P(3HB) was used for the first time by Hazari *et al.* [19] as a nerve conduit for a 10 mm gap in rat sciatic nerve regeneration. More studies on P(3HB) have been performed by another research team, where P(3HB) filled with alginate or alginate/fibronectin mixture was used to bridge a 1 cm gap of the rat sciatic nerve [20, 21]. Moreover, poly(3-hydroxybutyrate-*co*‐3‐hydroxyhexanoate), a copolymer was successfully used as a nerve conduit for repairing 10 mm defects in the sciatic nerve of the Sprague–Dawley rats [22]. Recently, not only scl-PHAs, but also mcl-PHAs have been investigated for their application in nerve regeneration, due to exhibiting mechanical properties closer to that of native nerve tissue, compared to P(3HB) and FDA-approved polymers PLLA, poly ε-caprolactone (PCL) and PLGA [3, 4, 14]. Homopolymers of poly(3‐hydroxyoctanoate), P(3HO), P(3HB) and their blends were developed and evaluated for their nerve regeneration [23, 24]. It was found that both neat polymers and their blends showed high cytocompatibility with respect to the NG108-15 neuronal cell line. The highest biocompatibility and neurite extension were achieved for the 25:75 blend of P(3HO)/P(3HB). In another study, P(3HO) has been studied *in vivo* for the production of a peripheral nerve graft [25]. P(3HO) tubes initiated the regeneration of axons and demonstrated good soft tissue response within 60 days. However, during the studied period, the nerve autograft control group demonstrated greater neuro-regeneration in comparison to the P(3HO). This is the only study evidenced using mcl-PHAs, in tubular form, for peripheral nerve repair.

Here, we expand the evaluation of mcl-PHAs as potential materials for peripheral nerve regeneration. Three mcl-PHAs namely poly(3-hydroxyoctanoate) (P(3HO)), poly(3-hydroxyoctanoate-*co*-3-hydoxydecanoate) (P(3HD-*co*-3HO)) and poly(3-hydroxyoctanoate-*co*-3-hydoxydecanoate-*co-*3-hydroxydodecanoate) (P(3HO-*co*-3HD-*co*-3HDD)) were assessed and compared with other synthetic and natural aliphatic polymers. Mechanical and thermal properties of these mcl-PHAs were characterized to evaluate their processability and potential as implantable materials in soft tissue engineering. Furthermore, the biocompatibility of these bio-polyesters was assessed with respect to NG108-15 neuronal cell line and rat primary Schwann cells. NG108-15 neuronal cell differentiation and rat primary Schwann cell phenotype were also assessed.

## Materials and methods

### Biodegradable aliphatic polymers

Microbial production of mcl-PHAs was conducted with the use of *Pseudomonas mendocina* CH50 (NCIMB 10541) and sodium octanoate, glucose [26] and coconut oil [17] as carbon source for the synthesis of P(3HO), P(3HD-*co*-3HO) and P(3HO-*co*-3HD-*co*-3HDD), respectively. P(3HB) was produced by *Bacillus subtilis* OK2 using glucose as the carbon source according to the procedure described [14]. Synthetic aliphatic polyester, PCL and poly(L-lactic acid) were supplied by Vornia Biomaterials (Ireland).

### Polymer sample preparation and characterization

Polymer film samples were prepared by the solvent-casting method as previously described [17, 23]. Briefly, polymers were dissolved in chloroform at 5 wt%, and 10 ml of polymer solution dispensed into 60 mm diameter glass petri dishes [17]. Solvent evaporation was completed in 5 days, in a fume cupboard at room temperature [23]. Samples were kept for 5 weeks at room temperature prior to their characterization.

For *in vitro* cell culture, solvent-cast films were punched into 13-mm^2^ circular samples using a biopsy punch. Samples were held in place, in 24 cell culture well plates, using its respective 10 wt% polymer solution, which acted as a glue [23, 27]. Sterilization of the films was conducted with 70% ethanol for 2 h, and subsequently washed twice with PBS, the latter remaining overnight ensured removal of alcohol residue [27]. Samples were rinsed once more in PBS before cell seeding.

DSC 214 Polyma (Netzsch, Germany) was used for the characterization of thermal properties of the polymers. Approximately 5 mg of polymer samples were sealed in pierced aluminium pans. Samples of mcl-PHAs of known history were used for thermal analysis [17]. The samples were subjected to the following thermal steps: first heating from −70 to 150°C (heating rate of 10°C per min); a 2-min isothermal step at 150°C; cooling to −70°C at 20°C per min; second heating to 150°C (heating rate of 10°C per min) [17, 23]. Proteus 7.0 software was used to determine glass transition, melting point and enthalpy of melting from DSC thermograms. A similar procedure was used for the characterization of other polyesters, however, the maximal temperature was 200°C [23].

A 5942 Testing Systems (Instron) was used for the characterization of film materials in tensile mode [23]. The instrument was equipped with 500 N load cell. All testing was conducted at room temperature. For tensile testing, strips of 5 mm width and length of 3.5–5.0 cm were cut from the cast film disc [17, 23].

### *In vitro* cell culture

#### NG108-15 neuronal cell culture

NG108-15 (ECACC 88112303) neuronal cells were cultured as previously described in supplemented Dulbecco’s modified Eagle’s medium (DMEM) containing 10% foetal calf serum [24, 27]. Passages 11–18 were used for the experiments. Thirty thousand cells were seeded onto each polymer film, topped up with DMEM and incubated at 37°C and 5% CO_2_ [24, 27]. After 2 days in culture, cultured medium was changed to DMEM containing no serum, to initiate the growth of neurites from NG108-15 neuronal cells [24, 27]. Experiments were completed after a total of 6 days in culture [24, 27].

#### Primary Schwann cell culture

Rat primary Schwann cells were harvested and expanded using the experimental methods as previously published [28]. Sciatic nerves, obtained from 10–12 week old adult male Wistar rats, were dissociated enzymatically and mechanically to release the Schwann cells [28]. Schwann cells were expanded in D-valine containing DMEM culture media to prevent fibroblast growth [28]. Once pure, 60 000 cells were seeded onto polymer films and left in culture for 6 days in the DMEM growth medium containing 10% foetal calf serum and 0.01% forskolin (Sigma Aldrich) [28]. Cells were cultured at 37°C with 5% CO_2_ and culture medium was changed after 3 days to replace nutrients [28].

#### Neuronal and Schwann cell viability

Viability of neuronal and Schwann cells, cultured on polymer films, was evaluated using the live/dead cell viability assay [24, 27]. Briefly, old cell culture medium was replaced with serum-free DMEM containing 0.001% Syto-9 (Invitrogen) and 0.0015% propidium iodide (Invitrogen), and incubated for 30 min at 37°C and 5% CO_2_ [24, 27]. Samples were imaged using an upright Zeiss LSM 510 confocal microscope and three fields of view were imaged per sample to calculate cell numbers (live versus dead cells) and by live/dead cell ratio as a cell viability percentage per polymer sample type [24, 27].

#### Immunolabelling of neuronal cells and Schwann cells

NG108-15 neuronal cells, and primary Schwann cells, were immunolabelled for specific proteins to visualize neurite outgrowth from neuronal cells and confirm Schwann cell phenotype, respectively, as previously described [24, 27]. Briefly, samples were fixed with 3.7% (v/v) paraformaldehyde (20 min), permeabilized with 0.1% Triton X-100 (20 min) and blocked with 3% bovine serum albumin (BSA), in PBS (30 min) [24, 27].

NG108-15 neuronal cells were labelled with a mouse anti-β III-tubulin antibody (1:250) (Promega, UK), for 48 h at 4°C, followed by a Texas Red-conjugated anti-mouse IgG antibody (1:200 dilution in 1% BSA from Vector Labs, USA), for 90 min at room temperature, to visualize and measure neurite outgrowth [24, 27].

Schwann cells were identified with a polyclonal rabbit anti-S100β (1:250) (Dako, Denmark) antibody, incubated for 48 h at 4°C, followed by a FITC-conjugated secondary anti-rabbit IgG antibody (1:100 dilution in 1% BSA), for 90 min at room temperature [24, 27]. All cells were incubated with 4,6-diamidino-2-phenylindole dihydrochloride (DAPI) (Sigma Aldrich) (300 nM) for 30 min, at room temperature to observe cell nuclei [27]. Cells, and samples, were imaged using an upright Zeiss LSM 510 confocal microscope using three fields of view for quantitative analysis [24, 27].

#### Neuronal cell differentiation and primary Schwann cell morphology assessment

As a proxy for nerve regeneration, neuronal cell differentiation was assessed quantitatively, measuring the percentage of cells expressing neurites; average number of neurites per neuron; average neurite length and maximum neurite length. Confocal images were quantified using Image J image processing program (NIH) [24, 27]. Neurites were traced, from the cell body to the neurite tip, and measured using the Neuron J plugin tracer software, measuring 90 neurites per polymer type [24, 27]. ITCN cell counter was used to count cells to quantify the percentage of cells bearing neurites and the average number of neurites per neuron. One hundred Schwann cells, were measured, length and width, with the ruler tool on NIH Image J to calculate average Schwann cell length and aspect ratio [27, 29].

### Statistical analysis

Statistical analysis was performed using a One-way analysis of variance (ANOVA; *P* < 0.05) incorporating Tukey’s multiple comparisons test if *P* < 0.05, using GraphPad Instat (GraphPad Software, USA) as previously described [24, 27]. Statistical analysis of the live/dead analysis was conducted using a two-way analysis of variance (*P* < 0.05) incorporating a Sidak’s multiple comparisons test if *P* < 0.05 [27]. A total of three independent experiments (*N* = 3) were performed for each assay, and images taken in triplicate (*n* = 3). Data were reported as mean±SD, *P* < 0.05 [24, 27].

## Results and discussion

### Thermal and mechanical properties of aliphatic polymers

**Figure 1.**
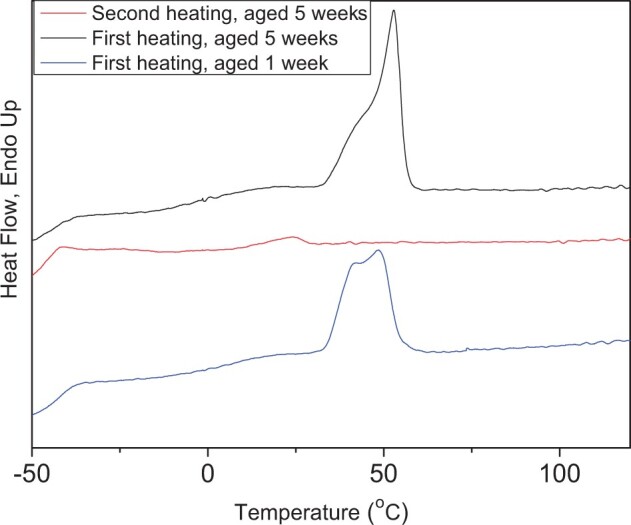
Representative DSC thermograms of P(3HO-*co*-3HD) aged for 1 and 5 weeks at room temperature. Samples of P(3HO-*co*-3HD) were heated from 70 to 150°C at a heating rate of 10°C per min [17, 23].

In this study, three mcl-PHAs are benchmarked against PCL and PLLA, two synthetic aliphatic polymers, which have been widely involved in biomedical research and used in some biomedical products. Additionally, P(3HB) has been included in the study, as the most studied representative of PHAs. Initial characterization was focussed on the most fundamental properties of materials, such as thermal and mechanical properties, which define processability and applicability of the materials. DSC revealed a very similar pattern in thermal response for all three mcl-PHAs. It is shown in Fig. 1 for P(3HO-*co*-3HD). DSC results for all other polymers used in this study are presented in online supplementary material (Supplementary Fig. S1). All mcl-PHAs exhibited low temperatures of glass transition and melting event in the temperature range between 35 and 60°C. Thus, all mcl-PHAs are semi-crystalline polymers.

The observed glass transition temperatures namely −38, −45.1 and −42.8°C for P(3HO), P(3HO-c*o*-3HD) and P(3HO-*co*-3HD-*co*-3HDD), respectively (Table 1), are in line with the published experimental results and modelled data for mcl-PHAs [30].

**Table 1.** Thermal properties of the polymers used in this study^a^

PHA	*T_g_* (^o^C)	End *T_m_* (^o^C)	Δ*H* (J/g)
P(3HO)	−38.0	58.8	20.5
P(3HD-*co*-3HO)	−45.1	56.4	19.3
P(3HO-*co*-3HD-*co*-3HDD)	−42.8	56.8	16.0
P(3HB)	n.d./3.8^b^	174.5	88.4
PLLA	n.d./51.3^b^	193.8	84.7
PCL	−60.0	69.4	79.0

a All results from first heating scan of samples aged at room temperature for 5 weeks.

b *T_g_* from the second heating (not detected (n.d.) in the first heating).

For mcl-PHAs, an increase in the length of side chains is expected to reduce glass transition temperature. As a result, glass transition temperature decreases from around 2°C for P(3HB) to −38°C for another homopolymer, P(3HO), and further reduction occurred for copolymers containing longer side chains. PCL is another polyester with low glass transition temperature (−60°C) [31]. On the contrary, glass transition temperature of PLLA is 51°C, and amorphous phase of this polyester occurs in a glassy state at room temperature.

In the characterization of the thermal properties of the polymers, it is a common practice to report results for thermal transition from the second heating scan when thermal history of a sample is erased. However, a distinctive difference between mcl-PHAs and other polyesters is the absence of endothermic peak in the second heating (Fig. 1 and Supplementary Fig. S1). Thus, crystalline phase did not develop when the mcl-PHA melt was cooled down (in our specific experiment at cooling rate of 20°C). This means that, under the conditions of the DSC experiments, the mcl-PHAs vitrified before crystallization. However, due to the low temperature of glass transition of mcl-PHAs, the amorphous phase is in a rubbery state at room temperature. Therefore, these polymers undergo slow crystallization during the storage and melting peak appears in the first DSC scan of aged samples. Thus, mcl-PHAs belong to a group of slow-to-crystallize polymers. Similar progress in crystallization occurs for the solvent-cast samples of all mcl-PHAs under study. As an example, Fig. 1 demonstrates that, in the first heating scan, the height of the melting peak for P(3HO-*co*-3HD) solvent-cast films increased with sample storage from 1 to 5 weeks after solvent evaporation was completed. Simultaneously, melting temperature shifted to higher temperatures with sample storage. Slow crystallization kinetics of mcl-PHAs need to be addressed in the research community to enable comparison of material properties. Reporting the thermal properties of mcl-PHA samples with known thermal history would be more informative than results obtained from the second scan. For all three mcl-PHAs used in this study, an enthalpy of melting stabilized after polymer storage at room temperature for 5 weeks indicating completion of the crystallization process. Therefore, thermal properties of all polymers were determined using samples stored for 5 weeks. The slow crystallization of mcl-PHAs must be also considered in the fabrication process of different devices from these materials. Some additives might be required for acceleration of the crystallization process to achieve peak product performance soon after manufacturing.

The melting of the crystallites of all mcl-PHAs is in a similar temperature range, which is comparable with the melting temperature range of PCL. Low melting temperature is beneficial for melt processing, decreasing polymer degradation and processing costs. The enthalpy of crystal fusion drastically decreased for aged mcl-PHA samples compared with P(3HB), a polymer of the same polyester family (Table 1). These results are not conclusive as to whether mcl-PHAs crystallize to a lower degree as compared to scl-PHAs, since the enthalpies of melting of 100% crystalline mcl-PHAs are not known. However, it has been established that methylene groups in the side chains of polymers decrease the enthalpy of melting of the polymer crystals [32]. A tentative value of the ethylene group contribution to the heat of melting is −0.7 kJ mol^−1^. Therefore, for P(3HO) with five methylene group in the side chain, they would decrease the enthalpy of melting by 3.5 kJ mol^−1^ compared with P(3HB). Since the molecular weight of the P(3HO) monomer unit is 142.2 g mol^−1^, the side chain of P(3HO) should decrease the enthalpy of melting of the fully crystalline polymer by 24.6 J g^−1^ from 146 J g^−1^ for P(3HB) [33]. This evaluation indicates that the crystallinity of P(3HO) was around 17%, while P(3HB) was ∼60%. Although some other factors might decrease the enthalpy of melting of 100% crystalline mcl-PHAs, it is unlikely that P(3HO) crystallizes to a similar extend as P(3HB). In line with additive group contribution, the observed enthalpy of melting of P(3HO-*co*-3HD), and P(3HO-*co*-3HD-*co-*3HDD), which contain longer side chains, further decreased when compared with P(3HO).

**Figure 2.**
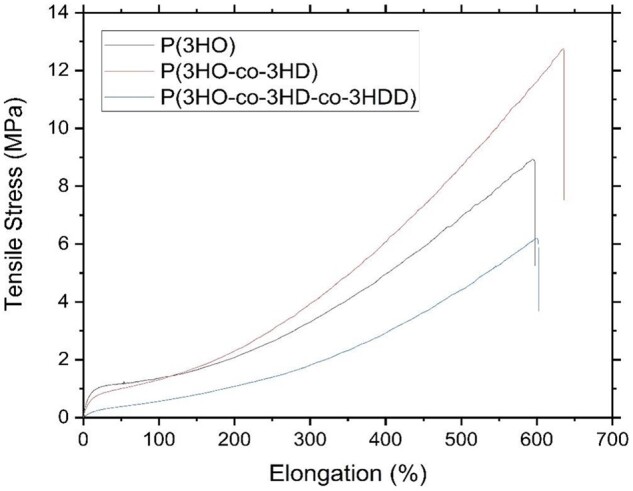
Representative stress–strain curves for mcl-PHAs: P(3HO), P(3HO-co-3HD) and P(3HO-co-3HD-co-3HDD) [24, 27].

A large fraction of the amorphous phase in mcl-PHAs and its rubbery state at room temperature are expected to define mechanical properties of these polymers. Figure 2 shows representative stress–strain curves obtained for these polymers in tensile testing.

All mcl-PHAs exhibited S-shaped stress–strain curves and ability to withstand large deformations (Table 2). After a narrow segment of elastic deformation, polymer stiffness gradually decreased. This was followed by a segment where a relatively small increase in stress induced large deformation. And finally, steep increase in stress–strain curves occurred at large deformations, which is characteristic of polymer stiffening with deformation. Such a response to deformation is characteristic for rubbery materials. Table 2 summarizes the mechanical properties of the polyesters. For mcl-PHAs, it contains results of modulus at 100% and 300% deformation (M100 and M300), parameters widely used in industries dealing with rubbers and elastomers. M100/M300 is tensile stresses at deformations of 100% and 300%, respectively. For all mcl-PHAs M300 was higher than M100.

**Table 2.** Mechanical properties of polymers^a^

	*σ_U_* (MPa)	*E* (MPa)	M100 (MPa)	M300 (MPa)	*ε_U_* (%)
P(3HO)	9.3 ± 0.7	14.1 ± 3.1	1.3 ± 0.15	3.3 ± 0.24	620 ± 30
P(3HD-*co*-3HO)	13.5 ± 1.1	10.2 ± 0.7	1.3 ± 0.2	3.9 ± 0.18	670 ± 50
P(3HO-*co*-3HD-*co*-3HDD)	6.6 ± 0.4	2.0 ± 0.3	0.56 ± 0.06	1.8 ± 0.11	540 ± 20
P(3HB)	21 ± 2	1300 ± 225	n/a	n/a	10 ± 1
PLLA	24 ± 1	1200 ± 50	n/a	n/a	2 ± 0.5
PCL	12.6 ± 0.5	270 ± 10	n/a	n/a	120 ± 70

a All results from the samples aged at room temperature for 5 weeks.

Despite similar pattern of stress–strain curves, mcl-PHAs exhibited some differences in their mechanical properties (Table 2). In the region of elastic deformation, P(3HO) was the stiffest material with elastic modulus (*E*) seven times higher than the softest P(3HO-*co*-3HD-*co*-3HDD). However, with the increase of deformation P(3HO-*co*-3HD) has the tendency to a larger stiffening. P(3HO-*co*-3HD-*co*-3HDD) was consistently softer than other two mcl-PHAs in the entire range of deformations before a break.

**Figure 3.**
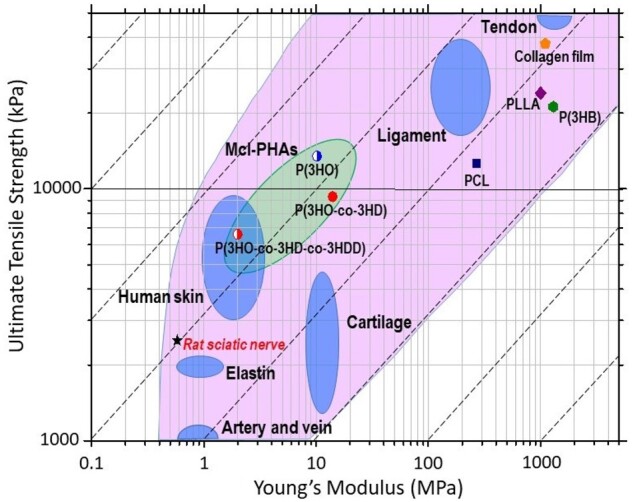
An Ashby plot of the tensile strength, of materials, plotted against Young’s modulus comparing the mechanical properties of polyesters with soft biological tissues. Adopted from (Ashby, 2008). Data for rat sciatic nerve and collagen film [34, 36].

Mechanical properties of other polymers used in this study are very different compared with the mcl-PHAs (Fig. 2, Table 2 and Supplementary Fig. S2). P(3HB) and PLLA are brittle and stiff materials. Only PCL was able to resist relatively large deformations, up to 120%. However, the stress–strain curve for PCL was distinctively different compared with P(3HB), PLLA and mcl-PHAs. Only the mcl-PHAs showed the stress–strain curve pattern similar to soft biological tissue. Most soft tissues including peripheral nerves are characterized by a J-shaped stress–strain curve [34, 35]. Such stress–strain curves are like the part of the S-shaped curve that followed after elastic deformation. Soft biological tissues do not have the initial segments of elastic deformation and decreasing stiffness of S-shaped curves due to a fact that they are pre-stressed materials. Thus, mcl-PHAs better emulate mechanical properties of soft tissues than other aliphatic polyesters. In order to demonstrate suitability of mcl-PHAs in applications for soft tissue regeneration, the Ashby plot containing data for soft tissues was populated with data for the polyesters used in this study (Fig. 3).

Mcl-PHAs are materials with the nearest mechanical properties to the peripheral nerves. Both parameters: tensile strength and modulus of mcl-PHAs exceeded the values for peripheral nerve. Of note, bulk collagen film properties, collagen an existing FDA-approved material for peripheral nerve repair, are located further from the peripheral nerve as compared to the mcl-PHAs [37, 38].

### *In vitro* analysis of mcl-PHAs using NG108-15 neuronal cells

**Figure 4.**
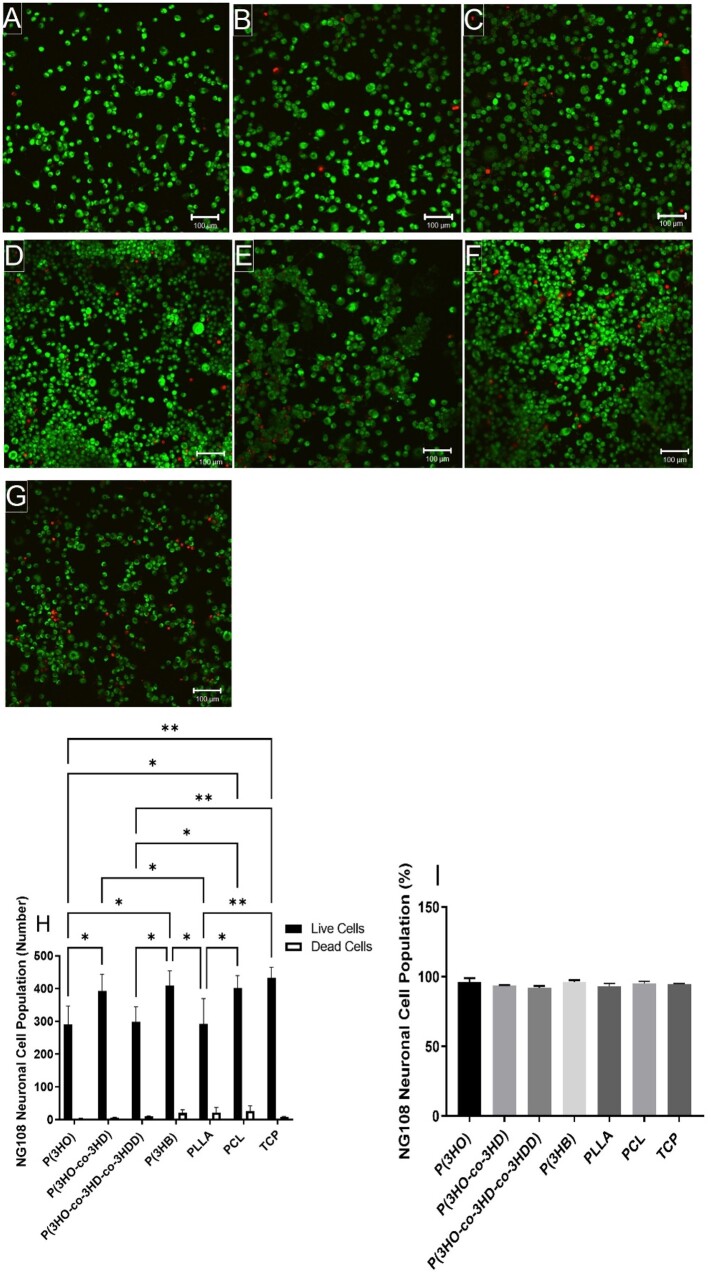
Confocal micrographs illustrating live (green) and dead (red) NG108-15 neuronal cells cultured on: (**A**) P(3HO) films; (**B**) P(3HO-*co*-3HD) films; (**C**) P(3HO-*co*-3HD-*co*-3HDD) films; (**D**) P(3HB) films; (**E**) PLLA films; (**F**) PCL films and (**G**) TCP control. Scale bar =100 μm. (**H**) Number of live cells versus dead cells per sample (mean ± SD, *n* = 3 independent experiments; *P < 0.05, and **P < 0.01) and (**I**) live cell viability as a percentage of live cells over total number of cells (mean ± SD, *n* = 3 independent experiments; *P* < 0.05) [24, 27].

NG108-15 neuronal cell biocompatibility, and viability, was assessed on solvent-casted films of P(3HO), P(3HO-*co*-3HD) and P(3HO-*co*-3HD-*co*-3HDD) and compared to other aliphatic polyesters, PCL, PLLA and P(3HB). Neuronal cells were labelled with syto 9 (live cell marker coloured green) and propidium iodide (dead cell marker coloured red) to visualize adhered cells and determine cell viability as a percentage. High numbers of live cells adhered to all surfaces with few dead, showing no signs of cytotoxic effect. Higher densities of live cells were observed on P(3HO-*co*-3HD), P(3HB) and PCL films (Fig. 4A, D and F, respectively).

When quantified (Fig. 4H), significantly higher numbers of live cells adhered to P(3HO-*co*-3HD), P(3HB) and PCL films, 393 ± 510, 409 ± 45 and 401 ± 38 cells, compared to the P(3HO), P(3HO-*co*-3HD-*co*-3HDD) and PLLA films, 291 ± 55, 298 ± 46 and 292 ± 48 cells,, respectively. P(3HO-*co*-3HD) films were superior at supporting NG108-15 neuronal cell adhesion, and maintaining cell growth, compared to the other mcl-PHAs, P(3HO) and P(3HO-*co*-3HD-*co*-3HDD). This results in the numbers of live neuronal cells comparable to PCL films, used in FDA-approved NGCs, as well as P(3HB) films. No significant differences were detected between experimental groups and all samples exhibited cell viability above 95%, deeming them biocompatible (Fig. 4I). This indicated that all polymer films promoted neuronal cell viability.

**Figure 5.**
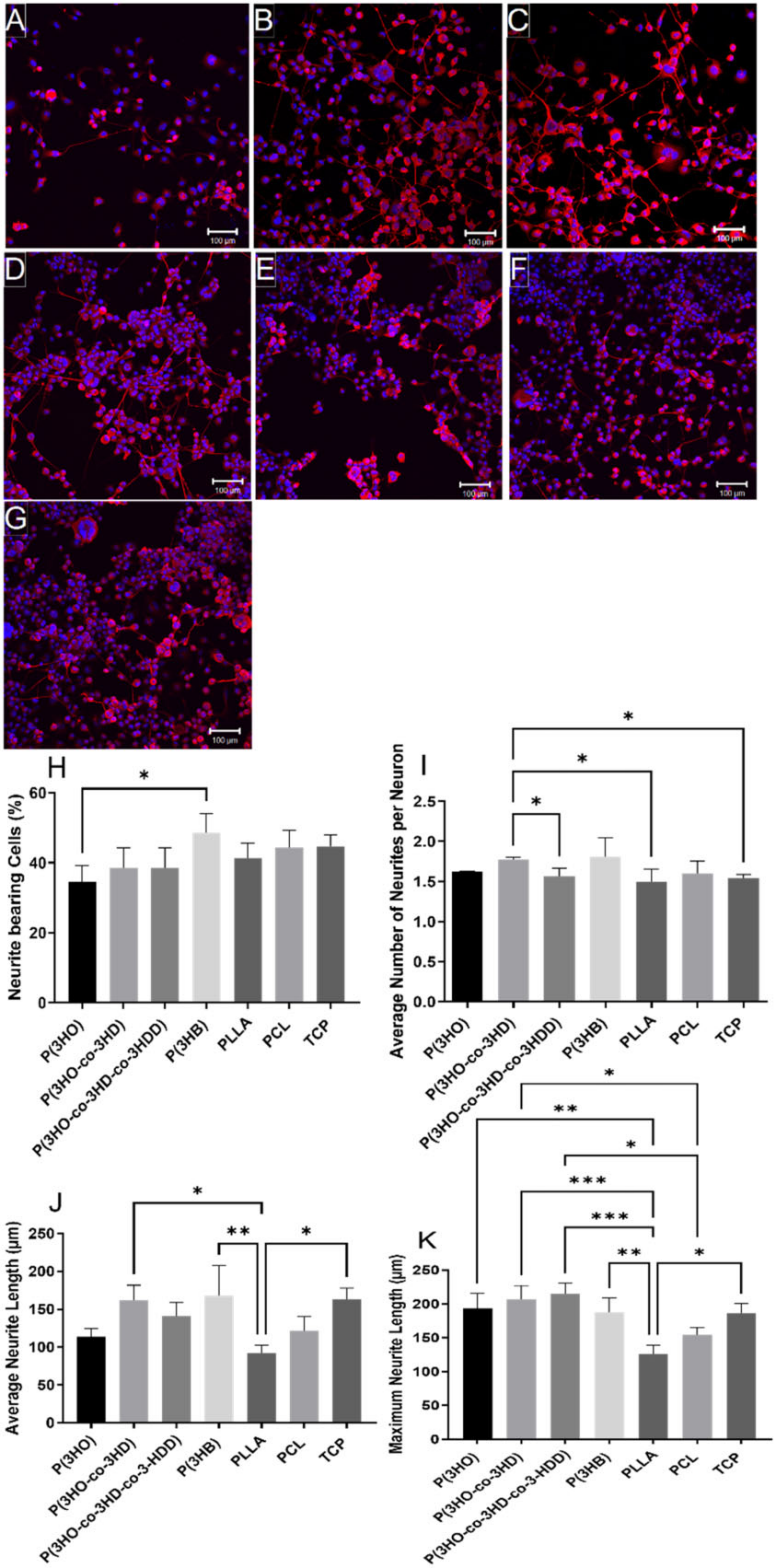
Confocal micrographs of NG108-15 neuronal cells immunolabelled for βIII-tubulin (red) and DAPI (blue) on: (**A**) P(3HO) films; (**B**) P(3HO-co-3HD) films; (**C**) P(3HO-co-3HD-co-3HDD) films; (**D**) P(3HB) films; (**E**) PLLA films; (**F**) PCL films and (**G**) TCP control. Scale bar =100 μm. (**H**) The percentage of neurite bearing neuronal cells (mean ± SD, *n* = 3; *P < 0.05); (**I**) the average number of neurites expressed per neuron (mean ± SD, *n* = 3; *P < 0.05) and (**J**) average neurite length per polymer condition (mean ± SD, *n* = 3; *P < 0.05 and **P < 0.01). (**K**) Maximum neurite length on all substrates (mean ± SD, *n* = 3; *P < 0.05, **P < 0.01 and ***P < 0.001) [24, 27].

To assess neuronal cell differentiation on polymer films, NG108-15 neuronal cells were labelled for neurite marker βIII-tubulin (red) and cell nuclei marker DAPI (blue) to label neurites and quantify cell differentiation. Neuronal cells adhered, and neurites could be visualized, on all polymer films and the TCP control (Fig. 5A–G), confirming optimal biocompatibility.

Confocal micrographs were quantified to determine neuronal cell differentiation by calculating the percentage of cells extending neurites (Fig. 5H), number of neurites outgrown per neuron (Fig. 5I), average neurite length (Fig. 5J) and maximum neurite length (Fig. 5K). In line with the cell viability assay, a significantly higher percentage of cells extending neurites was observed on the P(3HB) films 48 ± 5%, compared to cells cultured on the P(3HO) films, 35 ± 5%. No statistical differences were detected between other experimental conditions. A significant increase in the average number of neurites extending per neuron (Fig. 5I) was observed when neuronal cells were cultured on the P(3HO-co-3HD) films (1.8 ± 0.2) compared to cells cultured on the P(3HO-*co*-3HD-*co*-3HDD) films (1.6 ± 0.2), PLLA films (1.5 ± 0.2) and TCP control (1.5 ± 0.2). No significant differences were detected between the other datasets.

The average neurite length of neurites, outgrown from neuronal cells, cultured on the P(3HO-*co*-3HD), P(3HB) films and TCP control were 162 ± 20, 168 ± 40 and 163 ± 15 µm, respectively. These were significantly longer than those measured on the PLLA films, 92 ± 10 µm. No significant difference was detected between the other experimental conditions. Overall, the P(3HO-*co*-3HD) films supported growth of longer neurites compared to the other mcl-PHAs, P(3HO) and P(3HO-*co*-3HD-*co*-3HDD). With regards to maximum neurite length, significantly longer neurites were observed on all the PHA films, P(3HO-*co*-3HD), P(3HO-*co*-3HD-*co*-3HDD), P(3HO) and P(3HB), 207 ± 20, 215 ± 15, 193 ± 22 and 187 ± 20 µm, respectively, compared to the PLLA films, 126 ± 13 µm. Both P(3HO-*co*-3HD) and P(3HO-*co*-3HD-*co*-3HDD) films supported significantly longer maximum neurite lengths compared to neurites measured on the PCL films (154 ± 11 µm). The optimal levels of cell bearing neurites and number of neurites per neuron, as well as the neurite length displayed by the PHA films indicate that these substrates can maintain effectively the differentiated NG108-15 neuronal cells. This ability could favour the regrowth of axons in the proximal stump of injured peripheral nerves that leads the reconnection of the two nerve stumps [39]. However, as expected, the growth of NG108-15 neuronal cells on all these flat substrates was characterized by an irregular distribution of cells. This random migration indicates the importance of the addition of engineered guidance cues to direct cell migration ensuring an adequate nerve re-innervation when used as scaffolds in NGCs.

### *In vitro* analysis of mcl-PHAs using primary Schwann cells

**Figure 6.**
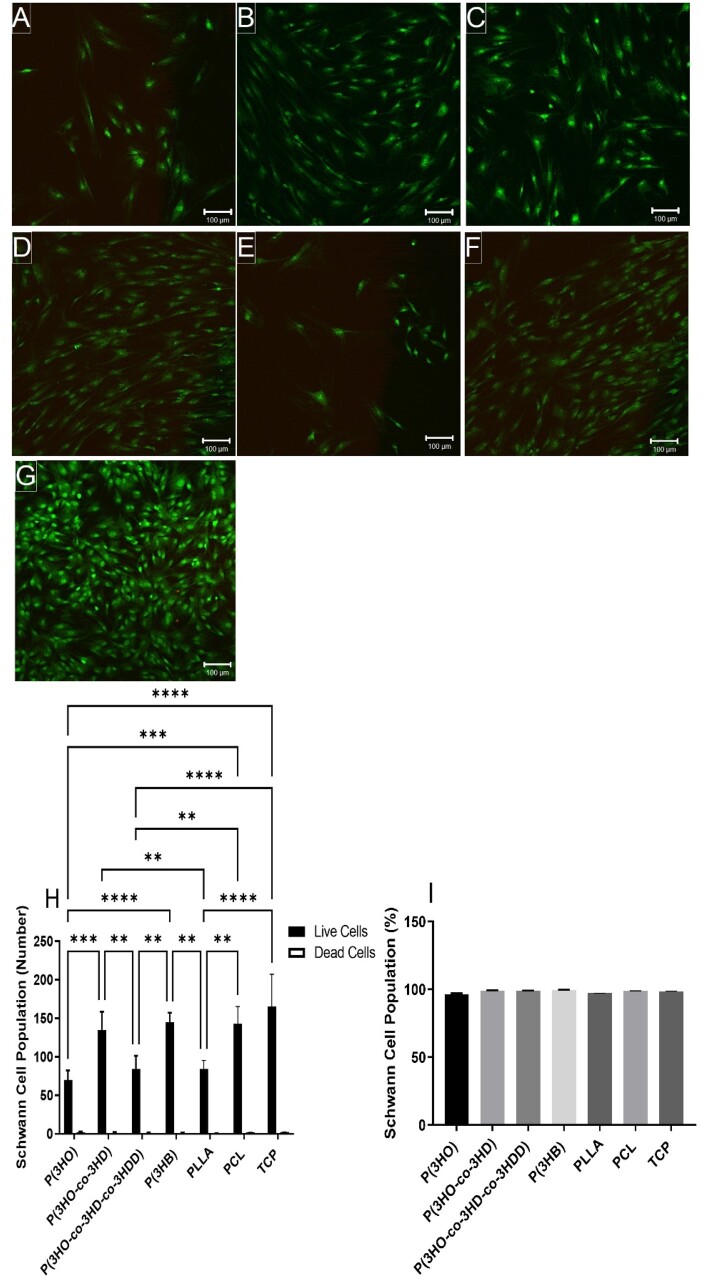
Confocal micrographs illustrating live (green) and dead (red) rat primary Schwann cells cultured on: (**A**) P(3HO) films; (**B**) P(3HO-*co*-3HD) films; (**C**) P(3HO-*co*-3HD-*co*-3HDD) films; (**D**) P(3HB) films; (**E**) PLLA films; (**F**) PCL films and (**G**) TCP control. Scale bar =100 μm. (**H**) Number of live cells versus dead cells per sample (mean ± SD, *n* = 3 independent experiments; *P < 0.05 and **P < 0.01) and (**I**) live cell viability as a percentage of live cells over total number of cells (mean ± SD, *n* = 3 independent experiments; *P* < 0.05) [24, 27].

Confocal micrographs were analysed to determine the cell viability of primary Schwann cells. Solvent-casted polymer films supported Schwann cell attachment, and little dead cells seen on the substrates (Fig. 6A–G).

The study of Schwann cells is relevant for neural repair approaches due to their crucial role in peripheral nerve regeneration after injury. Schwann cells promote a favourable environment for axonal regrowth via secretion of neurotrophic factors (such as nerve growth factor, brain‐derived neurotrophic factor and vascular endothelial growth factor), synthesize ECM molecules and express cell surface ligands [40]. Live Schwann cell attachment was greatest on the P(3HO-*co*-3HD), P(3HB), PCL films and TCP control. The P(3HO-*co*-3HD), P(3HB), PCL films and TCP control supported significantly higher numbers of adhered live Schwann cells, 135 ± 24, 145 ± 10, 143 ± 20 and 166 ± 40 cells,, respectively, compared to the P(3HO), P(3HO-*co*-3HD-*co*-3HDD) and PLLA films, 70 ± 10, 84 ± 17 and 85 ± 11 cells, respectively. No significant differences were detected between the P(3HO-*co*-3HD), P(3HB), PCL films and TCP control. All polymer films supported >95% cell viability, confirming once again their good biocompatibility. For both cell types, adhered cell population was higher on P(3HO-*co*-3HD), P(3HB) and PCL surfaces. This group of materials includes a soft P(3HO-*co*-3HD), very rigid P(3HB) and PCL with rigidity between P(3HO-*co*-3HD) and P(3HB). Similarly, the group of polymers with poorer cell adhesion includes materials of wide range of mechanical properties.

**Figure 7.**
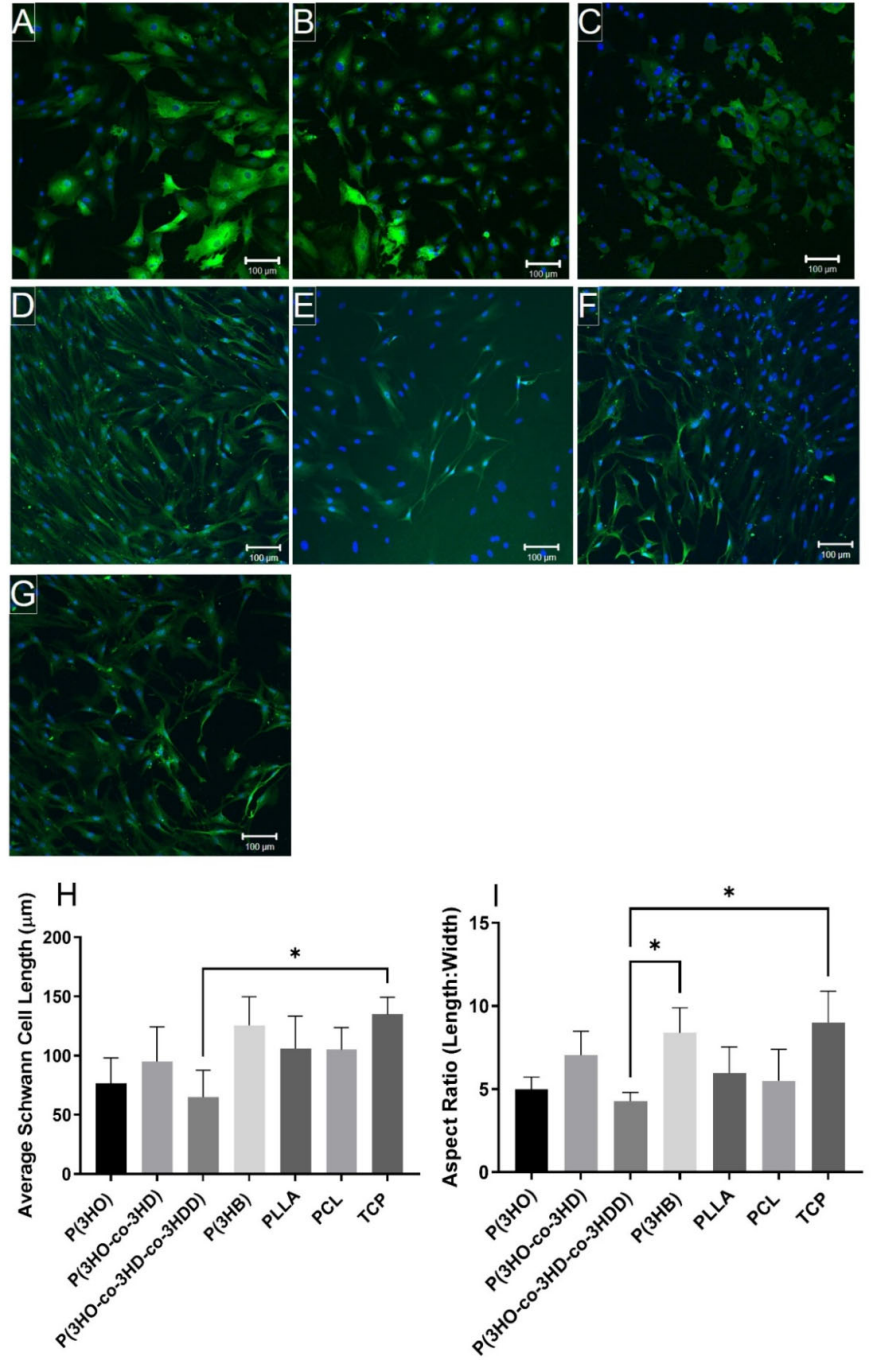
Confocal micrographs of rat primary Schwann cells immunolabelled for S100β (green) and DAPI (blue) on: (**A**) P(3HO) films; (**B**) P(3HO-*co*-3HD) films; (**C**) P(3HO-*co*-3HD-*co*-3HDD) films; (**D**) P(3HB) films; (**E**) PLLA films; (**F**) PCL films and (**G**) TCP control. Scale bar =100 μm. Schwann cell phenotype was assessed by calculating the (**H**) average Schwann cell length; and (**I**) aspect ratio (length/width) (mean ± SD, *n* = 3 independent experiments; *P < 0.05) [24, 27].

The effect of material type, on Schwann cell phenotype and morphology, was assessed by calculating the average Schwann cell length, as well as the aspect ratio (length: width) of cells [29]. Schwann cells stained positively for cytoskeleton marker S100β and attached to all surfaces (Fig. 7A–G).

The mcl-PHA films appeared to favour the multipolar morphology of Schwann cells, rather than the bipolar morphology, which was seen on the other films and TCP control. Schwann cells, with a significantly lower average length, were detected on the P(3HO-*co*-3HD-*co*-3HDD) films, 65 ± 22 µm, compared with the TCP control, 135 ± 14 µm (Fig. 7H). No other significant differences were detected between the additional polymer films. Similar results were observed when calculating the Schwann cell aspect ratio, which was significantly lower on the P(3HO-*co*-3HD-*co*-3HDD) films, 4.3 ± 0.5 µm, compared to the TCP control and P(3HB) films, 9 ± 2 and 8.4 ± 1.5 µm, respectively (Fig. 7I). Both, the morphology and the biochemical behaviour of Schwann cells appear to reflect their surrounding environment. *In vivo*, Schwann cells maintain their bipolar phenotype to function properly in that specific biomechanical environment along with the axons [41]. However, when Schwann cells are cultured *in vitro*, they display a range of motile phenotypes including unpolarized, bipolar or multipolar morphologies [5, 29], which could be clearly seen in Fig. 7A–G. The adoption of a particular phenotype can be influenced by the presence of other cells, such as neurons or specific cues present on the substrate. In the course of cell migration, the bipolar and multipolar phenotypes are involved in directional exploration and probing [42]. Moreover, the bipolar phenotype is known to be an essential feature of the motile and migratory Schwann cells [41]. Hence, as expected, the motile phenotypes unpolarized, bipolar and multipolar were observed in all the substrates analysed (Fig. 7A–G). Although a substantial number of bipolar Schwann cells were seen on the PCL and TCP, the predominant phenotype exhibited in all the substrates was multipolar. Unpolarized phenotype was abundant in mcl-PHAs films. In a similar study but using polymeric fibres, 1, 5, and 8 µm diameter PCL-aligned fibres displayed predominantly the bipolar morphology of Schwann cells grown in co-culture with neuronal cells [27]. It should be noted that the bipolar phenotype of Schwann cells is observed in the early stages of development of the PNS, in which the bipolar elongation processes along axons establishes the myelin internode [43]. Recently, neuronal cells have been shown are mechanosensitive in which Kayal *et al.* [44] reported that substrate stiffness could control NG108-15 cell neurite outgrowth directionality. However, P(3HO-*co*-3HD) exhibited similar neuronal and Schwann cell viability and differentiation properties P(3HB), which exhibit contrasting mechanical properties. Therefore, no correlation between mechanical properties and surface colonization with NG108-15 neuronal and primary Schwann cells was observed for these polyesters, and therefore likely due to differences in the materials, which is being investigated further.

Overall, mcl-PHAs widen the range of materials selection for soft tissue engineering applications, due to their thermal properties, mechanical properties as well as their biocompatibility, as shown by neuronal and Schwann cell viability, differentiation and phenotype. However, the mcl-PHAs exhibit low *T_m_* values and low *T_g_* values causing these polymers to be elastomeric and relatively sticky at room temperature. They are difficult to process using high temperature manufacturing methods, such as hot melt extrusion. Therefore, these materials have been blended with another PHA, P(3HB) and synthetic polymers, such as PLCL to manufacture tubular structures for nerve regeneration as described in Lizarraga-Valderrama *et al.* and Mendibil *et al.* to improve suitability for manufacturing [24–26]. The addition of mcl-PHAs, such as P(3HO) and P(3HO-*co*-HD), to harder, more brittle polymers, such as P(3HB), have been advantageously shown to reduce mechanical properties of the more brittle polymer, and improving mcl-PHA suitability for manufacturing, in recent studies, fabricating polymer fibres from electrospinning and pressurized gyration [24, 45] to dip coated tubular structures [46] and hot melt extruded tubes [26]. Blending of PHAs could be further exploited by the use of core-sheath technology, in which P(3HB), exhibiting superior biocompatibility for both NG108-15 neuronal cells and rat primary Schwann cells, could be used as sheath material, whereby the mcl-PHA, such as the P(3HO-*co*-HD), the core material providing more optimal mechanical properties for nerve tissue engineering applications [47].

## Conclusions

In summary, this study focussed on the comparative evaluation of three mcl-PHAs, P(3HO), P(3HD-*co*-3HO), P(3HO-*co*-3HD-*co*-3HDD), and other natural or synthetic aliphatic polyesters as biodegradable materials with potential applications in peripheral nerve regeneration. The mechanical, and thermal, properties of these mcl-PHAs widen the range of materials selection for soft tissue engineering providing materials enabling low temperature processing and mechanical properties closely matching nerve tissues. However, one must take into account the low *Tm* and *Tg* values exhibited by the mcl-PHAs when choosing appropriate processing methods. Although P(3HB), a rigid PHA, provided a superior substrate for supporting neuronal cells, the mechanical properties of this PHA limit its application in nerve regeneration. Therefore, to overcome the issues associated with processing the mcl-PHAs with high temperature methods, such as hot melt extrusion, they can be blended with P(3HB) to improve processability and biocompatibility. All mcl-PHAs demonstrated good attachment and viability of differentiated NG108-15 neuronal cells and primary Schwann cells, confirming their biocompatibility. Although P(3HB), a rigid PHA, provided a superior substrate for supporting neuronal cells, the mechanical properties of this PHA limit its application in nerve regeneration. Overall, P(3HO-*co*-HD) exhibited superior performance in supporting neuronal and Schwann cells viability among the mcl-PHAs evaluated. Moreover, this polymer was able to maintain differentiated neuronal and Schwann cells more efficiently than the other mcl-PHAs. Irregular distribution and orientation of NG108-15 neuronal cells were observed on all flat substrates under investigation signifying the importance of engineered guidance cues to direct axons for the appropriate generation of peripheral nerves. The applications of mcl-PHAs can be extended to the regeneration of other tissues with their use in multiphase polymer systems, such as polymer blends, e.g. with P(3HB) or polymer composites, which can potentially cover the whole range of mechanical properties from soft to hard tissues.

## Supplementary Material

rbad063_Supplementary_Data
