# Supplementary material for: Medium chain length polyhydroxyalkanoates as potential matrix materials for peripheral nerve regeneration
Source: Regen Biomater. 2023 Jul 21;10:rbad063. doi: 10.1093/rb/rbad063 (PMC10369215; doi:10.1093/rb/rbad063)
Supplement: rbad063_Supplementary_Data [file rbad063_supplementary_data.docx]

**Evaluation of medium chain length Polyhydroxyalkanoates as matrix materials for peripheral nerve regeneration**

R. Nigmatullin^1,2^, C.S. Taylor^3^, P. Basnett^2^, B. Lukasiewicz^2^, A. Paxinou^2,4^, L. Lizarraga-Valderrama^5^, J.W. Haycock^3^ and I. Roy^3^*

^1^ Higher Steaks Ltd., 25 Cambridge Science Park Road, Cambridge, CB4 0FW, UK

^2^School of Life Sciences, College of Liberal Arts and Sciences, University of Westminster, London, W1W 6UW, UK

^3^ Department of Materials Science and Engineering, The University of Sheffield, Sheffield, S3 7HQ, UK

^4^ Foundation of Research and Technology Hellas, Institute of Chemical Engineering and High Temperature Chemical Processes (FORTH/ICE-HT), P.O. Box 1414, GR 26504, Rion, Patras, Greece

^5^ School of Life Sciences, Queen's Medical Centre, University of Nottingham, NG7 2UH, UK

* Corresponding author:

**Figure S1**. Representative DSC thermograms of P(3HO) (a), P(3HO-*co*-3HD) (b), P(3HO-*co*-3HD-*co*-3HDD) (c), P(3HB) (d), PLLA (e), and PCL (f) aged for 5 weeks at room temperature.

**
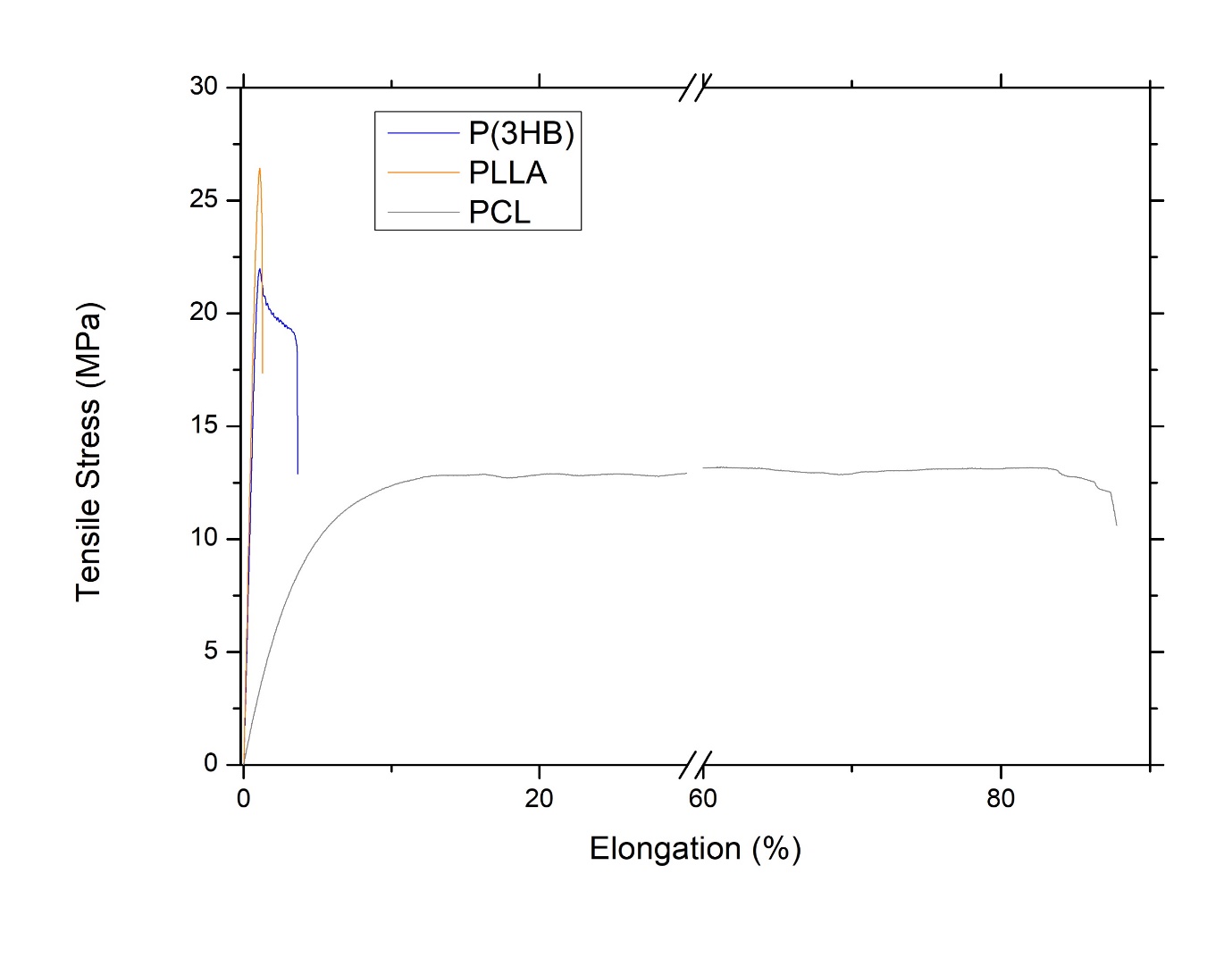
**

**Figure S2**. Representative stress-strain curves for P(3HB), PLLA, and PCL. Elongation interval between 30 and 60% is cut for better visualisation of curves for polymers with poor deformability (P(3HB) and PLLA) and stretchable PCL on the same graph.
